# Different Hyaluronic Acid‐Based Formulations Exert Composition‐Dependent Effects on Human Fibroblasts and Osteoblasts

**DOI:** 10.1111/jre.70094

**Published:** 2026-03-12

**Authors:** Caroline Viola Busch, Giulia Brunello, Kathrin Becker, Jürgen Becker, Elena Calciolari, Benedetta Ghezzi

**Affiliations:** ^1^ Department of Oral Surgery University Hospital of Düsseldorf Düsseldorf Germany; ^2^ Department of Oral Medicine, Infection, and Immunity Harvard School of Dental Medicine Boston Massachusetts USA; ^3^ Department of Orthodontics and Dentofacial Orthopaedics Charité ‐ Universitätsmedizin Berlin, Corporate Member of Freie Universität Berlin and Humboldt‐Universität Zu Berlin Berlin Germany; ^4^ Centre for Oral Clinical Research, Institute of Dentistry, Faculty of Medicine and Dentistry Queen Mary University of London London UK; ^5^ Centre of Dental Medicine, Department of Medicine and Surgery University of Parma Parma Italy; ^6^ IMEM‐CNR, Institute of Materials for Electronics and Magnetism‐National Research Council Parma Italy

**Keywords:** cellular proliferation, hyaluronic acid, octenidine, periodontitis therapy, polynucleotides, wound healing

## Abstract

Hyaluronic (HA)‐based materials show composition‐ and concentration‐dependent responses. Proper formulation and dosage selection are key for clinical application.

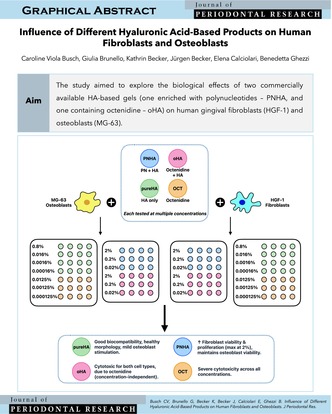

## 1 Introduction

Hyaluronic acid (HA) is widely used in dentistry for its role in wound healing [1], and hard [2] and soft tissue regeneration [3]. Several HA‐based formulations have been introduced aiming to enhance their therapeutic effects by accelerating wound healing [4] or by interfering with biofilm formation [5].

Despite the increasing availability of HA‐based products, there is still no conclusive evidence identifying the most effective formulation for specific indications, their interactions with different cell types involved in wound healing, as well as the contribution of the individual raw components.

Therefore, this study aimed to provide a head‐to‐head comparison on the biological effects of two commercially available HA‐based gels (one enriched with polynucleotides—PNHA, and one containing octenidine—oHA) on human gingival fibroblasts (HGF‐1) and osteoblasts (MG‐63).

## 2 Methods

PNHA (Regenfast, Geistlich Pharma AG, Wolhusen, Switzerland) and oHA (Pocket‐X, Geistlich Pharma AG) were tested at varying concentrations (0.02%, 0.2%, and 2%) to assess their effects on HGF‐1 and MG‐63 viability, metabolic activity, and morphology (details in Supporting Information). In addition, pureHA (Merck, Darmstadt, Germany) and octenidine, OCT (Octenisept, Schülke & Mayr GmbH, Norderstedt, Germany), were evaluated independently at concentrations corresponding to those present in the respective gel formulations, including a range of serial dilutions. Details are presented as Supporting Information.

## 3 Results

**FIGURE 1:**
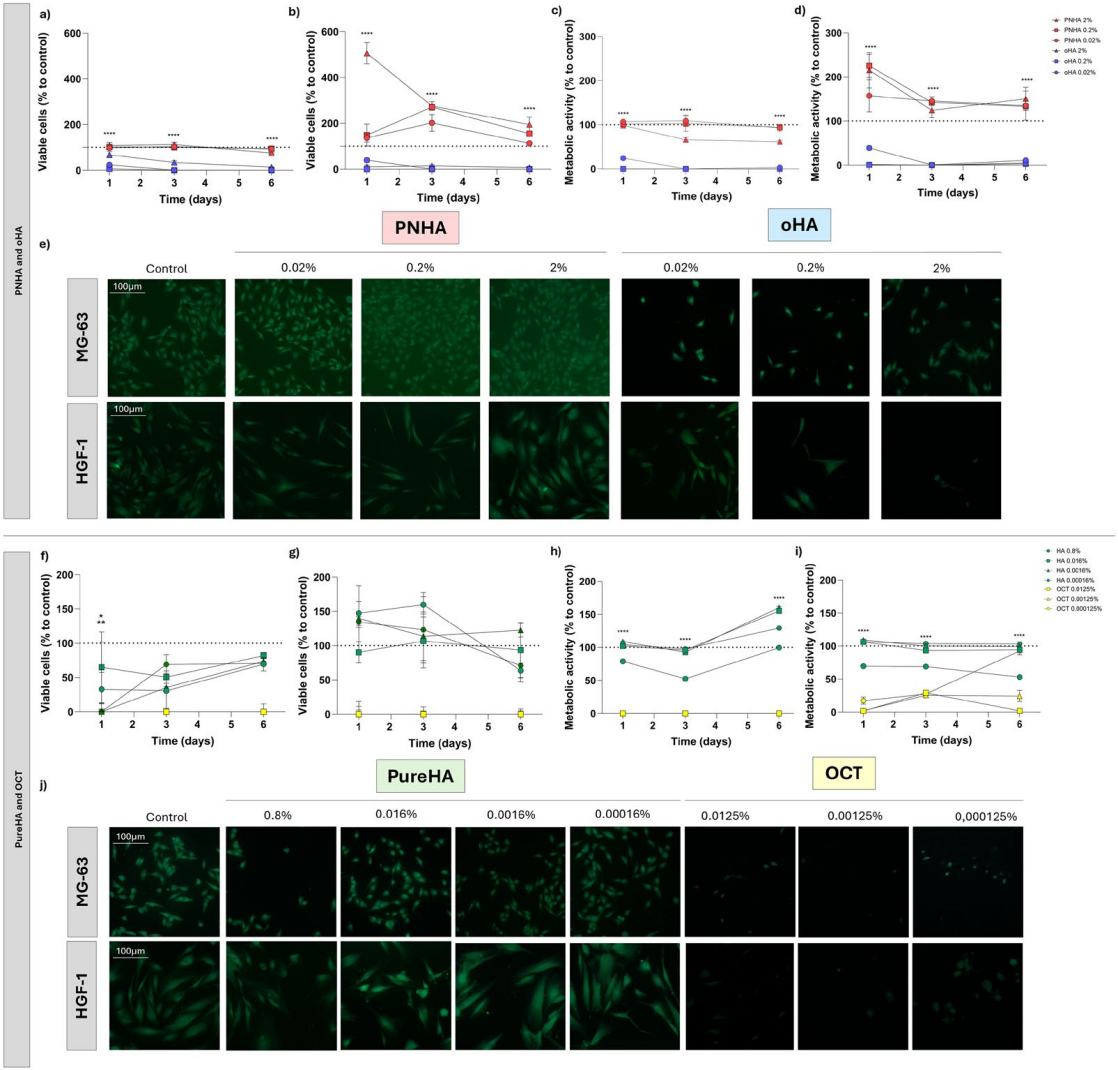
Viability assay and metabolic activity analysis of human osteoblasts (a–c), and gingival fibroblasts (b–d) cultured in the presence of PNHA and oHA; fluorescence images of MG‐63 and HGF‐1 cultured in the presence of PNHA or oHA (e). Viability assay and metabolic activity analysis of human osteoblasts (f–h), and gingival fibroblasts (g–i) cultured in the presence of pureHA or OCT; fluorescence images of MG‐63 and HGF‐1 cultured in the presence of pureHA or OCT (j). Data are expressed as mean values and standard deviations. Scale bar = 100 μm. *p < 0.05; **p < 0.01; ****p < 0.0001.

MG‐63 and HGF‐1 showed different viability patterns when cultured with PNHA and oHA (Figure 1a,b).

MG‐63 viability was comparable among all tested PNHA concentrations, for all experimental time points, while 2% PNHA positively influenced the initial activation of HGF‐1, with a higher viability still at T3 and T6 if compared to the 0.02% group. For both cell types, there was a marked difference between the two biomaterials tested at all the selected dilutions at T1, T3, and T6. By contrast, oHA groups showed a constantly low activity, thus suggesting a potential toxic effect of some components. Viability analysis findings are corroborated by proliferation and cell metabolic activity data (Figure 1c,d). The metabolism of both cellular lines maintained the same pattern over time and at the different concentrations of biologic factor. oHA negatively influenced the cells, while PNHA showed positive results, with significant differences between the two biomaterials at all time points and concentrations.

The analysis of the individual oHA components (Figure 1f–j), pureHA and OCT, at the same relative concentrations confirmed the initial findings, showing a marked toxic effect of pure OCT at all tested concentrations in both cell lines, with no significant differences. In the presence of pureHA, MG‐63 proliferation seems dose‐dependent at T1/T3; however, values converged at T6, although remaining lower than the control samples (reference value at 100%). Conversely, pureHA enhanced HGF‐1 proliferation during the culturing period, with viable cell numbers comparable to or higher than control.

Metabolic activity analysis revealed residual activity in OCT‐exposed cells, which progressively declined over time in parallel with increasing cell death, while pureHA treatment was comparable to the control, except for 0.8% pureHA, which was consistently lower (*p* < 0.0001) for both cell types.

Calcein‐AM staining (Figure 1e,j) confirmed viability and morphology outcomes. PNHA supported well‐adhered, healthy osteoblasts and fibroblasts, whereas oHA impaired cell adhesion and proliferation. Analysis of oHA components showed severe octenidine‐induced cytotoxicity, while pure HA preserved normal morphology, confirming its cytocompatibility.

## 4 Discussion

This in vitro study is the first direct comparison between two HA‐based commercial formulations that act by inducing distinct, concentration‐ and composition‐dependent responses in human fibroblasts and osteoblasts. PNHA supported fibroblast viability and metabolic activity, whereas oHA caused cytotoxicity. Even acknowledging the partial contribution of HA characteristics [6] (i.e., molecular weight, reticulation, additives, etc.), the obtained results highlight that commercially used HA‐based products are not biologically equivalent and provide the first dose‐dependent comparison in soft‐ and hard‐tissue cells. Consistently with previous reports [7], HA promoted soft tissues cells proliferation and migration. This is particularly evident when HA is combined with polynucleotides, confirming previous data of a synergistic effect of the two components, as PNHA activates pro‐survival and migratory signaling pathways in fibroblasts and epithelial cells [4, 7]. In line with this evidence, PNHA exhibited the most pronounced benefits at 2%, while osteoblastic responses remained largely limited over time. This confirms literature data [8] asserting HA supports osteoblastic viability while exerting only limited proliferative stimulation, and suggests that longer exposure or additional cues may be required for differentiation.

In contrast, oHA and OCT were highly cytotoxic. Although the antiseptic properties of oHA may be handy for the treatment of a chronic infection like periodontitis and its use has been successfully documented as adjuvant in pocket irrigation or as mouthwash to inhibit bacterial re‐colonization and facilitate plaque control [5], our results underscore that antiseptic‐containing formulations may hinder tissues healing.

Collectively, these results highlight that both formulation type and dosage are critical determinants of HA biocompatibility. Careful selection of HA‐containing biomaterials based on the specific clinical indications is therefore essential to maximize benefits and minimize adverse effects.

## 5 Conclusion

The results confirm that HA‐based products are not biologically equivalent. PNHA enhanced HGF‐1 and MG‐63 viability and proliferation in a concentration‐dependent manner, whereas oHA showed octenidine‐related cytotoxicity. This highlights the importance of concentration and formulation in biomaterial selection based on the clinical scenario and specific indication.

However, potential discrepancies between in vitro cytotoxicity and clinical performance cannot be ruled out when looking at factors such as dilution effects, tissue barriers, exposure time, and antimicrobial benefit/risk balance in vivo. Hence, future in vivo studies are needed to explore the behavior of HA‐based products in the wound healing environment.

## Author Contributions

**Caroline Viola Busch:** conceptualization, methodology, investigation, data curation, writing – original draft, supervision. **Giulia Brunello:** conceptualization, methodology, investigation, data curation, writing – review and editing, supervision. **Kathrin Becker:** conceptualization, formal analysis, resources, data curation, writing – review and editing, project administration. **Jürgen Becker:** resources, data curation, writing – review and editing, project administration. **Elena Calciolari:** conceptualization, investigation, resources, data curation, writing – review and editing, project administration. **Benedetta Ghezzi:** methodology, validation, formal analysis, investigation, resources, data curation, writing – original draft, supervision.

## AI Statement

This manuscript used NLP (ChatGPT) for proofreading an entirely human‐generated text, but no other use of AI was made.

## Funding

This research did not receive any specific grant from funding agencies in the public, commercial, or not‐for‐profit sectors.

## Conflicts of Interest

The authors declare no conflicts of interest.

## Supporting information


**Data S1:** Supplementary Information.

## Data Availability

All the data related to the present paper are available upon reasonable request from the corresponding author.
