# Supplementary material for: Different Hyaluronic Acid‐Based Formulations Exert Composition‐Dependent Effects on Human Fibroblasts and Osteoblasts
Source: J Periodontal Res. 2026 Mar 12;61(6):736–8. doi: 10.1111/jre.70094 (PMC13471751; doi:10.1111/jre.70094)
Supplement: Supplementary file 1 [file JRE-61-736-s001.docx]

**Supplementary material**

**MATERIALS AND METHODS**

**Biomaterials**

Both PNHA (Regenfast®, Geistlich Pharma AG, Wolhusen, Switzerland) and oHA (Pocket-X®, Geistlich Pharma AG) were tested at varying concentrations (0.02%, 0.2%, and 2%) to assess their effects on the viability, metabolic activity, and morphology of human gingival fibroblasts (HGF-1) and osteoblasts (MG-63). In addition, pureHA (Merck, Darmstadt, Germany) and OCT (Octenisept, Schülke & Mayr GmbH, Norderstedt, Germany) were evaluated independently at concentrations corresponding to those present in the respective gel formulations, including a range of serial dilutions. Specifically, pureHA was tested at 0.8%, 0.016%, 0.0016%, and 0.00016%, while octenidine was assessed at 0.0125%, 0.00125%, and 0.000125%.

**Cell culture**

*In vitro* cellular assays related to the biocompatibility of the selected biomaterials were performed using HGF-1 and MG-63 obtained from the Istituto Zooprofilattico Sperimentale della Lombardia e dell'Emilia Romagna (IZSLER, Brescia, Italy) and the American Type Culture Collection (ATCC, distributed from LGC Standards srl), respectively. Cells were cultured in complete Dulbecco's Modified Eagle Medium (DMEM) High Glucose (Dominique Dutscher, Alsace, France), implemented with 1% Penicillin/Streptomycin (Pen/Strep, Corning, Mediatech Inc., Manassas, VA, USA) and 10% Foetal Bovine Serum (FBS, Dominique Dutscher).

Cells were cultured in 48-wells treated plates (Corning®, Merck, Darmstadt, Germany) under standard conditions in an incubator at 37 °C, 95% humidity and 5% CO_2_. Upon reaching 70% of confluence_,_ HGF-1 were passaged using 0.25% Trypsin (25-053-CI, Corning) up to passage 7, while MG-63 were cultured up to passage 12. Subsequently, they were seeded at a density of 10,000 cells/ml for all the *in vitro* experiments.

Each experiment included a control group consisting of untreated cells. Pristine culture medium was supplemented every other day, without medium replacement, to prevent the removal of the bioactive products initially added.

**Cell viability**

The CyQuant cell proliferation assay kit (Thermo Fisher Scientific, Carlsbad, USA) was used to assess the cyto-viability of biomaterials dilutions on HGF-1 and MG-63 cell lines at 1 (T1), 3 (T3), and 6 (T6) days of culturing. At each experimental time point the media was removed, cells were washed in phosphate-buffered saline (PBS, SigmaAldrich, St. Louis, MI, USA) and frozen at -80°C. At the final time point, both the samples and the standards were incubated with CyQuant dye according to the manufacturer’s instructions. Fluorescence was measured and quantified at ex480/em520 nm using a microplate reader (Varioskan Lux, Thermo Fisher Scientific, Carlsbad, USA).

**Cell metabolic activity**

The biological response, in terms of metabolic activity, of HGF-1 and MG-63 in direct contact with the biomaterials was assessed by using the MTT assay (3-[4,5-Dimethylthiazol-2-yl]-2,5-diphenyltetrazolium bromide; Roche Diagnostics GmbH, Mannheim, Germany) on days 1 (T1), 3 (T3), and 6 (T6). Briefly, the culture supernatant was removed from the plate and 100 µl of fresh medium diluted with 10% of MTT labeling reagent was added to the plate, followed by incubation at 37°C for 4 hours. Subsequently, 100 µl of solubilization solution was added to each well and the plates were incubated overnight under standard culture conditions. At the term of the incubation, the absorbance of the formazan product was quantified at 620nm with a Multiskan® FC microplate reader (Thermo Fisher Scientific).

Prior to performing the assays, all wells were monitored throughout the culturing time using an inverted microscope (Nikon Eclipse TS100, Nikon, Tokyo, Japan). Images were taken to document cell morphology and disposition during the experimental time, besides corroborating the results of the biological tests.

**Cell morphology**

To evaluate cell morphology of viable cells fluorescent staining was performed at 24 h using Calcein-AM (Sigma-Aldrich, St. Louis, MI, USA). Briefly, the culture supernatant was discarded, cells were washed with PBS, and subsequently incubated with Calcein-AM 4 µM in PBS for 15 min at room temperature in the dark. After thorough PBS washing, specimens were mounted under glass cover slips using fluorescence mounting medium (Dako Cytomation Fluorescence Medium, Agilent Technologies, Santa Clara, CA, USA) to minimize photo bleaching. Samples were observed with a fluorescence microscope Zeiss Axio Imager A.2 (Carl Zeiss, Jena, Germany) equipped with a 10X objective.

**Statistical analysis**

All values are presented as mean and standard deviation (SD) from a minimum of three independent measurements, each performed in triplicate. After the initial validation of the normal distribution of the residual values through Shapiro-Wilk test, the qualitative differences of the gel formulations (PNHA, oHA) at varying concentrations were evaluated in terms of cell viability and cell metabolic activity on MG-63 and HGF-1 cells using 2-way analyses of variance. The same analysis was repeated for pure products (pureHA, OCT). In case of significance, it was followed by the Tukey post-test for multiple comparisons. Results were considered statistically significant at p < 0.05. Data were analysed using Prism 10 (GraphPad Software, La Jolla, CA, USA).
